# 

**Published:** 1909-12

**Authors:** 


					11
DECEMBER, 1909.
Vol. XXVII. No. 106.
THE
BRISTOL
,?CHIRURGICAL
mj K IN \
S. G n
PUBLISHED UNDER THE AUSPICES ' w-
THE BRISTOL MEDICO-CHIRURG1Cj1LSWjTWy\
Editor: R. SHINGLETON SMITH, M.D., B.Sc.
WITH WHOM ARE ASSOCIATED
J. MICHELL CLARKE, M.A., M.D.,
J. LACY FIRTH, M.S., M.D., JAMES SWAIN. M.S., M.D.
P. WATSON WILLIAMS, M.D., Assistant Editor.
Editorial Secretary: J. A. NIXON, B.A., M.B.
BRISTOL: J. W. ARROWSMITH.
London : J. & A. Churchill, 7 Great Marlborough Street.
Price One Shilling and Sixpence,

				

